# Intraoperative tissue classification methods in orthopedic and neurological surgeries: A systematic review

**DOI:** 10.3389/fsurg.2022.952539

**Published:** 2022-08-03

**Authors:** Aidana Massalimova, Maikel Timmermans, Hooman Esfandiari, Fabio Carrillo, Christoph J. Laux, Mazda Farshad, Kathleen Denis, Philipp Fürnstahl

**Affiliations:** ^1^ Research in Orthopedic Computer Science (ROCS), Balgrist Campus, University of Zurich, Zurich, Switzerland; ^2^ KU Leuven, Campus Group T, BioMechanics (BMe), Smart Instrumentation Group, Leuven, Belgium; ^3^ Department of Orthopaedics, Balgrist University Hospital, University of Zurich, Zurich, Switzerland

**Keywords:** tissue classification, intraoperative, artificial intelligence, orthopedic surgery, neurological surgery, systematic review

## Abstract

Accurate tissue differentiation during orthopedic and neurological surgeries is critical, given that such surgeries involve operations on or in the vicinity of vital neurovascular structures and erroneous surgical maneuvers can lead to surgical complications. By now, the number of emerging technologies tackling the problem of intraoperative tissue classification methods is increasing. Therefore, this systematic review paper intends to give a general overview of existing technologies. The review was done based on the PRISMA principle and two databases: PubMed and IEEE Xplore. The screening process resulted in 60 full-text papers. The general characteristics of the methodology from extracted papers included data processing pipeline, machine learning methods if applicable, types of tissues that can be identified with them, phantom used to conduct the experiment, and evaluation results. This paper can be useful in identifying the problems in the current status of the state-of-the-art intraoperative tissue classification methods and designing new enhanced techniques.

## Introduction

Identifying and differentiating individual tissue types and anatomical structures is one of the most fundamental skills a surgeon must have to perform surgery in a safe and successful manner. Taking spinal surgery as an example, the combination of poor visualization, complex anatomy, and vital adjacent structures such as the spinal cord, peripheral nerves, and the aorta imposes a particular set of challenges for the surgeon (1). During spinal instrumentation, pedicle screws must be placed inside a safe corridor enclosed within the pedicles of each vertebra. Surgeons use their tactile and auditory senses along with their visual perception to determine anatomic landmarks and a specific tissue type (e.g., cortical bone vs cancellous bone) and avoid screw perforation. Differentiation between tissue types is particularly challenging in minimal invasive surgeries (MIS), such as percutaneous screw placement, due to the lack of direct visual information and unobscured haptic feedback (2). Depending on the incorporated surgical technique and the assessment metric, screw perforations can occur in 5% to 41% in the lumbar spine and from 3% to 55% in the thoracic region (3) which can cause serious peri- and postoperative complications (4). Accurate and real-time intraoperative tissue classification can help surgeons to identify whether a given pedicle screw is breaching (or has breached) the cortical bone layer and therefore alleviate potential malposition and the associated postoperative complications.

Computer-assisted surgical navigation can be noted as the most common technique for providing surgical guidance during operation (5, 6) and relies on either an optical or an electromagnetic tracking sensor to provide the positional information of surgical tools relative to the anatomy. The image-guided methods can provide intraoperative navigation and differentiation between rigid tissue types based on the registration of preoperative imaging data (e.g., computed tomography or magnetic resonance imaging) to the anatomy based on the real-time tracking information of the patient and the surgical instruments. Navigation techniques have been shown to result in superior implantation accuracy for the spine surgery use case (7). Similarly, the clinical usefulness of surgical navigation has been acknowledged in neurosurgery procedures (8). Owing to the registration process, the established navigation systems are, however, limited in their capability to differentiate tissues in real-time, especially soft tissue structures that deform during manipulation, as well as changes due to patient positioning.

Inspired by the enhanced ability of surgeons when combining visual and non-visual senses to determine a specific tissue type, a growing body of research strives to leverage intraoperative sensing technologies (e.g., hyperspectral imaging and vibro-acoustic sensing) for tissue classification and differentiation to improve the effectiveness of surgical navigation and autonomy of surgical robotics (9–12). Particularly, many of the emerging technologies provide tissue classification through utilizing non-visual sensing by either complementing the existing visual navigation systems to improve their accuracy or offering new functionalities and abilities for surgeons to monitor the surgical procedure. This way, registration of preoperative data to the anatomy can be rendered obsolete, which can potentially facilitate the adoption of such algorithms for intraoperative use.

The increasing number of publications on this topic suggests the need for a systematic review of available intraoperative tissue classification methods in the fields of orthopaedics and neurosurgery and to understand the development status of available technologies. In this article, we have characterized the existing papers with respect to the underlying sensing technology, the clinical application, the data processing algorithms, the classification accuracy, and outlook from a clinical perspective.

## Methods

### Search strategy

**Figure 1:**
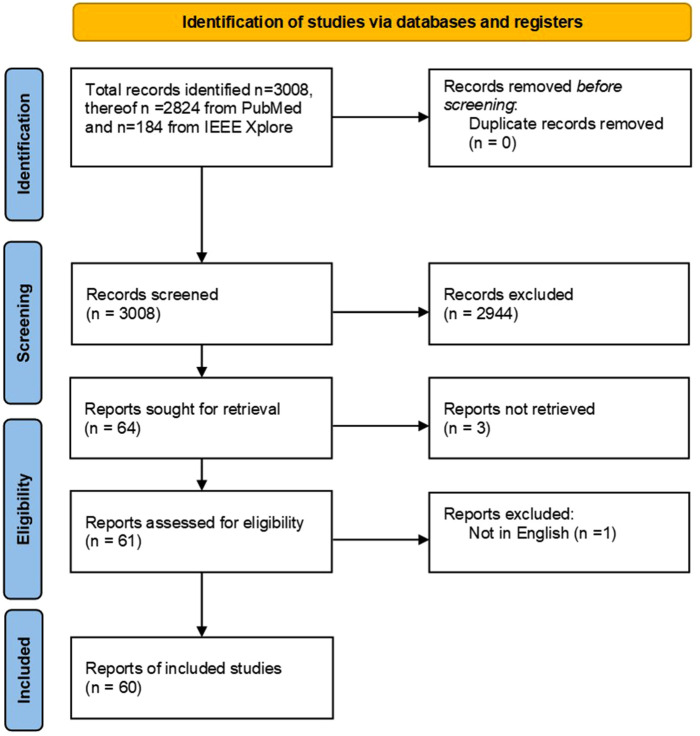
Prisma flow diagram.

In this study, we conducted a systematic review on intraoperative tissue classification methods in orthopedic and neurological surgeries based on the Preferred Reporting Items for Systematic Reviews and Meta-Analyses (PRISMA) guidelines (13). The search strategy is illustrated in the form of a flow diagram (Figure 1). Since the scope of this paper was on preclinical and clinical studies, PubMed was selected as the primary publication database and search engine. Additionally, IEEE Xplore was used to find relevant papers with a technical focus. The following search term was used in both databases:

(tissue OR nerve OR cartilage OR bone OR artery OR ligament OR state) AND (classif* OR sens* OR detect*) AND (surgery OR intraoperative) AND (orthop* OR spine OR neurosurgery) AND (data fusion OR sensor fusion OR machine learning OR deep learning OR artificial intelligence OR neural networks OR signal processing)

where “*OR”* and “*AND”* terms denote the corresponding logical operations between the individual keywords. The first group includes the tissue types such as nerve, artery, cartilage, bone, or ligament. The term “state” was added to include relevant articles concerning state identification within the anatomy. The second group comprises synonymous words for classification including sensing and detection. To include both nouns and gerunds, only the word root was used together with wildcards, e.g., classif* instead of classification and classifying. Since we intended to cover sensor fusion, machine learning, and classical signal processing, a third group was added accordingly.

### Study selection

Preliminary individual search and screening were performed on November 23, 2021, by two independent reviewers in a blinded fashion (Figure 1). In case of contradicting results, joint discussions between the two individual reviewers were conducted to reach a consensus. The screening was conducted based on the titles and the abstracts of the collected articles. A publication was deemed eligible to be included in this review if:

• The topic of the paper concerned real-time tissue classification during orthopedic and neurological surgeries.
• The method for classifying different tissue structures was binary or multi-class. Since some studies involved shape detection (e.g., x-ray-based vertebrae boundary detection), one versus rest classification approach-based reports were excluded.
• The study was either *in-vivo* or *ex-vivo* conducted on either human or animal tissues or synthetic anatomical models.

We only included studies that were published in English.

### Data extraction

To facilitate the systematic extraction of data from the included articles, we created the following template based on which the key characteristics of each study were recorded:

• The surgical task for which the proposed method was developed.
• The main sensor or sensing technology used for tissue classification.
• The implemented data processing steps.
• If the method involves machine learning: the name of the machine learning method or framework used.
• The type of specimen used in the study (e.g. human brain, animal brain, synthetic phantom, etc.)
• Classes or tissue types classified with the proposed method (e.g. tumor, blood vessels, cortical bone, etc.)
• Criteria used for performance evaluation such as accuracy, recognition rate, success rate, sensitivity, and specificity.
• Further prospective on clinical applications.

## Results

### Studies included

A total of 3,008 publications were retrieved through our initial database search. As no duplicates were found, two independent reviewers screened the titles and the abstracts of all retrieved publications. The screening process resulted in 2,944 papers that were further evaluated against the above-mentioned criteria. A total of 64 papers passed through the screening from which 60 full-text papers were included in this systematic review. Three papers were excluded due to missing online full text and one paper was excluded as it was not written in English.

The identified papers were grouped into categories depending on the sensing technology used, namely hyperspectral imaging, spectroscopic sensing, ultrasound imaging, force, robotic and impedance sensing, vibro-acoustic sensing, optical coherence tomography, endoscopic and microscopic imaging, and x-ray imaging. The general overview of categories is tabulated in Table 1.

**Table 1:** General overview of sensing technologies.

Category	Sensors	Surgical tasks	Classes
Hyperspectral imaging (9, 14–18)	VNIR hyperspectral pushbroom camera, NIR hyperspectral pushbroom camera, VNIR hyperspectral snapshot camera	brain tumor resection	healthy tissue, tumor, blood vessels, dura mater
Spectroscopic sensing (10, 11, 19–33)	optical scattering spectroscopy probe, Raman spectrometer, diffuse reflectance spectroscope, stimulated Raman scattering microscope, red and infrared lasers, Narrow-band imaging, coherent anti-stokes Raman scattering microscope, visible-resonance Raman spectrometer, laser displacement sensor, Q-switched frequency-doubled Nd:YAG and Er:YAG lasers, endo-microscope, fluorescence imaging and color imaging	brain tumor resection, minimally invasive spinal surgery, robotic laser-based orthopedic surgery, robotic orthopedic surgery, intracranial microsurgery, tumor resection,robotic bone milling, bone cutting	brain, nerve, fat, artery, muscle, solid tumor, infiltrating tumor, necrosis, normal tissue, bone, intervertebral disc, spinal cord, cartilage, subchondral, meniscus, cancellous bone, normal tissue, lesional tissue, low-grade tumor, high-grade tumor, malignant gliomas, diffuse lower-grade gliomas, pilocytic astrocytoma, ependymoma, lymphoma, metastatic tumor, medulloblastoma, meningioma, pituitary adenoma, gliosis, white matter, grey matter, nondiagnostic tissue, ligament, internal carotid artery, facial nerve, glioblastoma, melanoma, breast cancer, vertebrae, adjacent bony structures, hard bone, soft bone, skin
Ultrasound imaging (12, 34, 35)	Ultrasound, elastography	trans-psoas surgery, brain tumor resection	nerve, bone, psoas muscle, glioblastoma, solitary brain metastases, tumor, healthy tissue
Force, robotic control, and impedance sensing (36–46)	Custom-made mechatronic bone drilling tool, force sensor, current sensor, UR5 robotic arm, custom-made tactile sensing probe using balloon expansion, load cell, DC drill motor, robot manipulator, optical tracking sensor, impedance spectroscopy device	bone drilling, robotic bone drilling, robotic bone milling, tumor resection	breakthrough from cortical to cancellous bone, cortical bone, 30pcf cancellous bone, 50pcf cancellous bone, outer cortical bone, cancellous bone, inner cortical bone, white matter, gray matter, pedicle cortical bone, pedicle cancellous bone, vertebral cancellous bone, cortical transit cancellous, almost break cortical, fat, muscle fiber
Vibro-acoustic sensing (10, 47–57)	Laser displacement sensor, accelerometer, free-field microphone, inertial measurement unit chip, condenser microphone, contact microphone, non-contact acoustic microphone, sound recorder	robotic bone milling, robotic bone drilling, bone drilling, bone cutting, bone milling	vertebrae, spinal cord, adjacent bony structure, muscle, cortical bone, cancellous bone, annulus fibrosus, cortical bone, fascia, fat, liver, muscle, breakthrough, skin, outer cortical layer, inner cortical layer
OCT imaging (58–61)	OCT system, full-field swept-source OCT system	Brain tumor resection, tumor resection	Non-cancerous tissue, glioma-infiltrated tissue, vital tumor, healthy tissue, necrosis, meningioma, cortex, hippocampus, corpus callosum, striatum, thalamus
Microscopic and endoscopic imaging (62–66)	Surgical microscope, surgical endoscope	Spinal endoscopic surgery, percutaneous transforaminal endoscopic discectomy, craniotomy, microvascular decompression	Nerve, dura mater, vessel, parenchyma, trigeminal nerve, facial nerve, glossopharyngeal nerve, vagus nerve, anterior inferior cerebellar artery, –posterior inferior cerebellar artery, petrosal vein
X-ray imaging (67, 68)	X-ray	bone tumor resection	Benign tumor, malignant tumor, normal tissue

### Hyperspectral imaging

**Figure 2:**
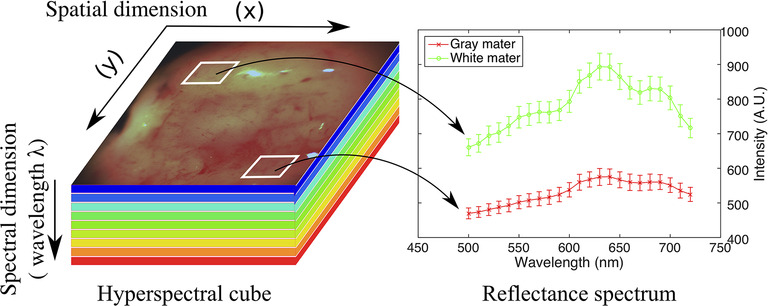
Spectral signatures generation for the specific tissue type (69).

Hyperspectral imaging is a spectroscopy method that captures both spectral and spatial information. Each pixel represents hundreds of spectral bands, which correlate with the chemical composition of the underlying imaged material (Figure 2).

#### Clinical application

Six publications (9, 14–18) were found in this category that focused on tissue classification based on hyperspectral imaging (Table 2). These articles focused on detecting brain tumor tissue since hyperspectral imaging facilitates non-contact, non-ionizing, and real-time data acquisition that is used for tumor diagnosis.

**Table 2:** General descriptions of selected studies on hyperspectral imaging.

Citation	Surgical Task	Sensor	Preprocessing	ML Method	Material	Classes	Evaluation
Fabelo *et al.* 2018	brain tumor resection	VNIR hyperspectral pushbroom camera	image calibration, image denoising, band removing, spectral averaging, normalization, dimensional reduction	SVM + KNN + HKM	brain tissue	healthy tissue, tumor, blood vessels, background	Acc.87.27%–100%
							Spec. 99.52%–100%
							Sens. 97.95%–100%
Fabelo *et al.* 2019a	brain tumor resection	VNIR hyperspectral pushbroom camera	image calibration, image denoising, image normalization	U-Net + 1D DNN + 2D CNN	brain tissue	healthy tissue, tumor, blood vessels, background	Acc. 0.78–0.81
Fabelo *et al.* 2019b	brain tumor resection	VNIR hyperspectral pushbroom camera	image calibration, noise filtering, band averaging, normalization	2D-CNN, PCA + SVM + KNN,1D DNN, SVM	brain tissue	healthy tissue, tumor, blood vessels, background	Acc. 84%–85%
							Sens. 25%–99%
							Spec. 90%–99%
							AUC 0.82–1.00
Leon *et al.* 2021	brain tumor resection	VNIR and NIR push-broom hyperspectral cameras	noise filtering, band removing, NIR upsampling, VNIR-NIR registration, cropping, spectral fusion, NIR reflectance offset adjustment	K-means, K-medoids, HKM, SVM, RF, KNN	synthetic phantom	material, color, material-color	Acc. 76.50%–90.6%
							Jaccard 0.53–0.76
Manni *et al.* 2020	brain tumor resection	VNIR hyperspectral pushbroom camera	image calibration, noise filtering, band removing ant colony optimization	3D-2D CNN	brain tissue	healthy tissue, tumor, blood vessels, background	Acc 0.80
							Sens. 0.68–0.96
							Spec. 0.87–0.98
							AUC 0.70–0.91
Urbanos *et al.* 2021	brain tumor resection	VNIR hyperspectral Snapshot camera	calibration, spectral correction, normalization	SVM, RF, 3D CNN	brain tissue	healthy tissue, tumor, venous blood vessel, arterial blood vessel, dura mater	Acc. 60%–95%

#### Technologies used

Extracted reports used different technologies of varying spectral wavelengths. The majority of studies used Visual and Near-Infrared (VNIR) push-broom cameras covering a 400–1,000 nm wavelength range (9, 14, 15, 17, 18). In contrast to push-broom imaging technology, Urbanos et al. (16) used a VNIR snapshot camera that can be used in a surgical environment due to its smaller footprint. However, such sensors generally deliver a narrower wavelength range, namely 655–955 nm. Leon et al. (14) proposed the combination of VNIR and Near-Infrared (NIR) cameras to cover the broadband spectral range between 435 nm and 1638 nm.

#### Data processing

The data preprocessing steps for hyperspectral imaging conventionally start with image calibration, where the raw data is calibrated with respect to white and dark reference images. This is followed by image denoising and band averaging. Afterward, spectral signatures are normalized to prevent the differences in radiation intensities caused by surface irregularities (9, 15–17). Unlike other studies, Leon et al. (14) additionally included a coarse-to-fine search algorithm, which was applied to gray-scale images from VNIR and NIR cameras to identify the most relevant bands for spatial registration. Feature-based registration technique using Speeded Up Robust Features (SURF) detectors with projective transformations was applied afterward to register two images.

The processed hyperspectral data is then generally used as an input for machine learning methods that are trained to perform tissue classification. Within the collected articles, different machine learning algorithms were used for this task, namely: Support Vector Machines (SVM) (14, 16, 17), K-Nearest Neighbors (KNN) (14), (Hierarchical) K-Means (HKM) (14), Random Forests (RF) (14, 16), 1-Dimensional (1D) Deep Neural Networks (DNN) (17), 2D Convolutional Neural Networks (CNNs) (17) and 3D CNNS (16). Fabelo et al. (15) proposed a more complex learning framework incorporating both supervised and unsupervised machine learning methods. First, an SVM classifier was employed to generate a supervised pixel-wise classification map. This was followed by fixed reference t-stochastic neighbors embedding dimensionality reduction algorithm and a KNN filtering in order to reach spatial homogenization. Secondly, a segmentation map was obtained by employing the unsupervised clustering method HKM. The two branches were combined with a majority voting approach (15).

#### Tissue classes and accuracy

The majority of studies were based on human brain tissue samples and aimed at differentiating between healthy tissue, tumor, blood vessels, and background using push-broom hyperspectral sensors. The method reported in Urbanos et al. (16) additionally classified venous, arterial blood vessels, and dura mater by a snapshot camera by conducting two evaluations, namely intra-patient and inter-patient. Experiments in Urbanos et al. (16) resulted in the highest overall accuracy of 95% using RF in an intra-patient evaluation. However, the inter-patient evaluation based on SVM showed a poor performance (acc. 60%), which was explained by the lower number of bands available through snapshot cameras and inter-patient variability (16). Fabelo et al. (15) achieved a robust classification using a hybrid network integrating supervised and unsupervised learning methods with an accuracy higher than 87.27%. After optimizing the algorithm, the total processing time was reported to be in the order of 1 min (15). The results of Leon et al. (14) revealed that the average classification accuracy after data fusion from VNIR and NIR cameras was 21% higher than when using individual camera data using SVM, KNN, and RF algorithms.

#### Outlook

In spine surgery, hyperspectral imaging might be useful in differentiating intervertebral disc or scar tissue from nerve structures to identify vital structures earlier, more accurately, and without missing sequestered disc fragments in close relation to other tissues. In this regard, till today there is no intraoperative technology providing that functionality.

### Spectroscopic sensing

Spectroscopy provides a precise analytical method for identifying the chemical composition of the material based on its emission spectrum. The contactless nature of the spectroscopic technology makes it suitable for intraoperative tissue identification during surgical interventions. Spectroscopy provides a similar reflectance spectrum as hyperspectral imaging but it does not capture spatial information. Instead, light is dispersed with diffraction grating first and then projected onto charge-coupled devices (CCDs). Afterward, spectral information is extracted from this digital format.

#### Clinical application

The majority of studies that we found in this category (19–28) used spectroscopic methods for delineating the brain tumor regions (Table 3) by showing that the spectroscopic information can successfully characterize tumor tissues during neurosurgery. Furthermore, Kenhagho et al. (29) demonstrated that optoacoustic properties can characterize the tissue type during laser osteotomy. Gunaratne et al. (30) showcased the feasibility of integrating diffuse reflectance spectroscopy into robotic knee laser surgery, which typically lacks tactile feedback.

**Table 3:** General descriptions of selected studies on spectroscopy.

Citation	Surgical task	Sensor	Preprocessing	ML Method	Material	Classes	Evaluation
Andrews *et al.* 2000	brain tumor resection	optical scattering spectroscopy probe	N/A	N/A	rat brain tissue	brain, nerve, fat, artery, muscle	N/A
Broadbent *et al.* 2018	brain tumor resection	Raman spectrometer	open morphology weighted penalized least square, third-order smoothing, band removing, normalization, median filtering, synthetic minority oversampling	SVM	brain tissue	solid tumor, infiltrating tumor, necrosis, normal tissue	Acc. 89%, Sens. 94%, Spec. 97%
Cakmakci *et al.* 2020	brain tumor resection	high resolution magic angle spinning nuclear magnetic resonance spectrometer	left shifting, frequency domain transformation, phase correction, cropping	PLS-DA, RF, SVM, NN, CNN	brain tissue	tumor/healthy tissue, benign/ malignant tumor	AUC 0.74–0.98
Chen *et al.* 2016	minimally invasive spinal surgery	Raman spectrometer	wavelet denoising, spike removing, standard normal variate, fluorescence background correction	PCA-LDA	swine backbone	bone, fat, intervertebral disc, muscle, spinal cord	Acc. 93.1%, Spec. 85.7%–100%, Sens. 82%–100%
Gunaratne *et al.* 2019	robotic laser-based orthopedic surgery	diffuse reflectance spectroscope	normalization, dimensionality reduction	LDA	joint tissue	cartilage, subchondral, meniscus, and cancellous bone	Acc. 99%, Sens. 99.2%–100%
Hollon *et al.* 2018	brain tumor resection	stimulated Raman scattering microscope	N/A	RF	brain tissue	normal/ lesional tissue, low-grade/ high-grade tumor	Acc. 89.4%–93.8%, AUC 0.96–0.97
Hollon *et al.* 2020	brain tumor resection	stimulated Raman scattering microscope	N/A	Inception ResNet- v2	brain tissue	malignant gliomas, diffuse lower grade gliomas, pilocytic astrocytoma, ependymoma, lymphoma, metastatic tumor, medulloblastoma, meningioma, pituitary adenoma, gliosis, white matter, grey matter, nondiagnostic tissue	Acc. 86.4%, IoU 0.51–0.93
Laws *et al.* 2020	robotic orthopedic surgery	red and infrared lasers	image cropping, resizing	GoogLeNET	shoulder sample	cartilage, ligament, muscle, metal surgical tools	Pre. 84.8%–100%, Re. 90.7%–100%
Livermore *et al.* 2020	brain tumor resection	Raman spectrometer	signal-to-noise thresholding, cosmic ray removing, third-order polynomial baseline correction, normalization	PCA-LDA	brain tissue	glioma, normal brain	Sens. 0.96, Spec. 0.99, Acc. 0.99
Puustinen *et al.* 2020	Intracranial microsurgery	custom-built narrow-band imaging	normalization, band reduction, Savitzky-Golay filter	U-Net	cadaveric temporal bone	internal carotid artery, facial nerve	Acc. 90%
Riva *et al.* 2021	brain tumor resection	Raman spectrometer	band reduction, median filtering, signal-to-noise ratio thresholding, background signal subtraction, outlier removal, signal normalization	RF, GB	brain tissue	glioma, normal brain tissue	Acc. 80%–83%, Pre.79%–82%, Re. 80–82%, F1 80%–82%, AUC 0.80–0.82
Uckermann *et al.* 2014	tumor resection	Coherent anti-Stokes Raman scattering microscope	N/A	N/A	brain tissue, breast tissue, mouse tissue	Glioblastoma, melanoma, breast cancer	N/A
Zhou *et al.* 2019	brain tumor resection	Visible Resonance Raman spectrometer	baseline removal, normalization	PCA-SVM	brain tissue	healthy brain tissue, normal control tissues, glioma tumors at low grades, glioma tumors at high grades	Acc. 53.7–96.3%, Sens.100%, Spec. 71%
Dai *et al.* 2015	robotic bone milling	laser displacement sensor	median filter, WPT	three-layer backpropagation neural network	porcine spine	vertebrae, spinal cord, adjacent bony structure, muscle	Suc. rate 83–100%
Kenhagho *et al.* 2021	bone cutting	Q-switched frequency-doubled Nd:YAG laser, Er:YAG laser	FFT, bandwidth selection	PCA+ quadratic SVM/gaussian SVM/ three-layer back propagation neural network	porcine proximal, distal femurs	hard bone, soft bone, muscle, fat, skin tissues	Err. 0–94.40%
Kamen *et al.* 2016	Brain tumor resection	clinical endo-microscope	Entropy-based image pruning, local features (Scale Invariant Feature Transform), feature coding (Bag of Words, sparse coding, locality-constrained sparse coding), feature pooling	SVM + majority voting	brain tissue	glioblastoma, meningioma	Acc. 0.83–0.84, Sens. 0.87–0.89, Spec. 0.77–0.81
Shen *et al.* 2021	bone tumor resection	Second near-infrared fluorescence imaging and color imaging combined instrument	White light (WL) image, fluorescence light (FL) image, normalization	FL-CNN, WL-CNN	brain tissue	tumor/ non-tumor	Spec. 0.803–0.822, Sens. 0.821–0.938, PPV 0.889–0.910, NPV 0.699–0.872, F1 0.853–0.824

#### Technologies used

Spectroscopic sensing methods found in the reviewed articles can be categorized into the following technologies: optical scattering spectroscopy probe (19), high-resolution magic angle spinning nuclear resonance spectrometer (21), Raman spectrometers (i.e. coherent anti- Stokes Raman scattering, visible resonance Raman, stimulated Raman scattering) (11, 20, 22–27), diffuse reflectance spectroscopy (30, 31), selective narrow-band imaging (32), clinical endomicroscopy (28), and fluorescence imaging (33). The largest subgroup among these studies was based on Raman Spectroscopy (RS). Raman spectra are generated from the interaction of the monochromatic laser beam radiation with the molecules of the tissue. The reflected light characterizes the contents of lipids, protein, and nucleic acid of the tissue under investigation. Shen et al. (33) presented a combination of fluorescence imaging and color imaging cameras to delineate brain tumor regions. This technology was capable of capturing white light and fluorescence images simultaneously. Q-switched frequency-doubled Neodymium-doped Yttrium Aluminum Garnet (Nd:YAG) and Erbium-doped Yttrium Aluminum Garnet (Er:YAG) lasers were compared during laser bone cutting in Kenhagho et al. (30).

#### Data processing

The preprocessing pipeline of spectral data consisted of signal denoising (11, 20, 24, 25), band reduction to extract biologically relevant spectra (20, 30, 32), normalization (20, 25, 27, 30, 32, 33), and median filtering to remove outliers (10, 20, 25). The majority of the proposed classification methods for Raman spectroscopy were based on traditional machine learning approaches including SVM (20), combined linear discriminant analysis (LDA) and principal component analysis (PCA) (11), combined SVM and PCA (27, 29), RF (22, 25), and gradient boosting (25). Hollon et al. (23) implemented a more complex learning method based on the inception ResNet-v2 network. Dai et al. (10) presented laser displacement sensor application during robotic bone milling based on wavelet packet transformation and artificial neural network. Gunaratne et al. (30) and Laws et al. (31) applied diffuse reflectance imaging in robotic orthopedic surgery with LDA and GoogLeNET.

#### Tissue classes and accuracy

Multiple studies aimed at using RS in detecting different types of tumor tissues such as solid tumors (20), infiltrating tumors (20), or glioma tumors with different gradings (22, 25, 27). Moreover, Hollon et al. (23) expanded the number of classes to 13 diagnostic tumor classes. The ground truth labels were based on conventional hematoxylin and eosin-staining histology results of the samples collected. The overall accuracy using inception ResNet-v2 was 86% and comparable with other methods (23). Articles related to orthopedic surgery reported the recognition of different tissue types including bone, fat, intervertebral disc, muscle, spinal cord, cartilage, and ligament. Gunaratne et al. (30) classified four different tissue types including cartilage, subchondral bone, cancellous bone, and meniscus tissues within the wavelength range of 200–1030 nm using LDA. The methodology achieved a better than 99% accuracy. Laws et al. (31) showed that an infrared laser is more accurate (acc. 97.8%) in classifying the shoulder joint tissue type (cartilage, ligament, muscle, and metal surgical tools) than a red laser (acc. 94.1%) using GoogLeNET. Chen et al. (11) used RS with a 785–1,100 nm spectral range to differentiate bone, fat, muscle, intervertebral disc, and spinal cord of the swine backbone. The overall accuracy of the proposed method was above 93.1%. Dai et al. (10) presented a success rate above 83% using a three-layer back propagation neural network in identifying vertebrae, spinal cord, adjacent bony structures, and muscle. Moreover, Nd:YAG and Er:YAG lasers were used to recognize hard and soft bone, muscle, fat, and skin from fresh porcine femurs during laser bone cutting in Kenhagho et al. (29). The amplitude of the signal generated with the Nd:YAG laser was greater than Er:YAG laser, which in turn contributed to the lower overall classification error. In terms of classification methods, the three-layer back propagation neural network outperformed SVM with an error rate of 5.01 ± 5.06% and 9.12 ± 3.39% using the Nd:YAG and Er:YAG lasers respectively (29). The fluorescence imaging-based deep learning approach outperformed the clinicians in terms of sensitivity, specifically 93.8% vs. 82% (33).

#### Outlook

Spectroscopic sensing is a potentially powerful technology for spine surgical use cases as it might assist in breach detection during pedicle screw preparation, detection of low-grade infections, differentiation of seroma and cerebrospinal fluid, and tissue differentiation.

### Ultrasound imaging

Ultrasound imaging is an established clinical imaging method that provides real-time information about anatomy and physiology based on the emission and detection of sonar signals. This technology has been widely used in guiding interventional and diagnosis procedures owing to its portability, low price, and non-invasiveness (no radiation exposure). However, it is typically accompanied by low resolution, image artifacts, and operator dependency.

#### Clinical application

Within the reviewed articles, three studies (12, 34, 35) unveiled the potential of ultrasound imaging for tissue classification (Table 4). Carson et al. (12) tackled the problem of nerve localization and identification in lateral lumbar interbody fusion surgery with a trans-psoas approach, where the disc space is accessed through the psoas muscle. Cepeda et al. (34) and Ritschel et al. (35) used ultrasound sensing in detecting brain tumor regions for tumor resection. Cepeda et al. (34) compared B-mode and elastography ultrasound techniques in detecting the brain tumor region. Ritschel et al. (35) used an additional contrast agent, namely a bolus injection, to detect the brain tumor during tumor resection.

**Table 4:** General descriptions of selected studies about ultrasound.

Citation	Surgical Task	Sensor	Preprocessing	ML Method	Material	Classes	Evaluation
Carson *et al.* 2021	trans-psoas surgery	ultrasound imaging system	tapered windowing function, time gain compensation, brightness normalization, dilation and erosion	U-Net	porcine tissue	nerve, bone, psoas muscle	Dice 83.81%–90.60%, Sens. 100%, Spec. 93.13% –98.61%, Acc. 96.30% – 98.29%
Cepeda *et al.* 2020	Brain tumor resection	Ultrasound, elastography	normalization, despeckling, Gaussian blur filter	inception V3 (transfer learning) + LR, SVM, RF, NN, kNN	brain tissue	glioblastoma, solitary brain metastases	AUC 0.791–0.985, Acc. 74.9%–94.7%, F1 0.724-0. 947, Pre. 0.779-0. 947, Re. 0.749-0. 947
Ritschel *et al.* 2015	Brain tumor resection	ultrasound system	N/A	SVM	brain tissue	tumor, healthy tissue	Pre. 0.71, TN 0.93, Acc. 0.90, Sens. 0.76, Spec. 0.94

#### Technologies used

Carson et al. (12) used an ultrasound imaging system (SonoVision,TM Tissue Differentiation Intelligence, USA). Cepeda et al. (34) reported using the Hitachi Noblus with a C42 convex probe, 8–10 MHz frequency range, 20 mm scan width radius, and 80° scan angle of field of view. In Ritschel et al. (35), the ultrasound system Aplio XG 500 (Toshiba Medical Systems, Japan) in the second harmonic imaging mode was used along with a 2 ml of contrast agent SonoVue (Bracco Imaging, Germany). By sweeping over the skin surface of the anatomy, one can visualize the underlying anatomical structures using an ultrasound system. Ultrasound waves are transmitted from the transducer to the anatomy. The ultrasound images are formed based on the echoes received and the time duration to receive the signal back. The echoed signal differs depending on the sensed tissue type.

#### Data processing

In the work by Carson et al. (12), the edges of the B-mode images were suppressed with tapered windowing functions followed by time gain compensation and normalization. Then, the morphological operations including dilation and erosion were applied. Processed images were used further to train a U-Net architecture. Cepeda et al. (34) cropped and removed small peripheral artifacts from B-mode images and the corresponding areas from elastograms. Afterward, images were rescaled, normalized, despeckled, and smoothed with Gaussian filtering. The transfer learning method using the inception V3 network was used with the preprocessed data. Then, classification methods such as logistic regression, SVM, RF, neural network, and KNN were applied. Ritschel et al. (35) differentiated tumors from healthy tissue with SVM.

#### Tissue types and accuracy

The data collection for the U-net method was conducted on in-vivo porcine specimen with the objective to categorize it into three classes, namely nerve, bone, and psoas muscle (12). The ground truth labels were created by clinical experts. The network demonstrated high performance and achieved a 83.81%-90.60% dice score, a 100% sensitivity, a 93.13%–98.61% specificity, and a 96.30%–98.29% accuracy across all three classes (12). Other approaches based on ultrasound addressed brain tumor resection (34, 35) as a binary classification problem of glioblastomas vs. solitary brain metastases and tumor tissue vs. healthy brain tissue. Elastography (acc. 79%–95%) based algorithms outperformed B-mode (acc. 72%–89%) in Cepeda et al. (34). Ritschel et al. (35) reached a 90% accuracy and a 76% sensitivity.

#### Outlook

With accurate real-time imaging of soft tissues even with plastic deformation, ultrasound offers a potential benefit for anterior approaches to the spine, which are challenging due to their proximity to vital soft-tissue structures, especially in the revision situation with scarring and altered anatomy. Furthermore, by visualizing posterior vertebral structures, ultrasound could be used as a data basis for non-ionizing spinal navigation.

### Force, robotic, and impedance sensing

Through physical contact and tactile sensation, humans can perceive a variety of object properties including size, hardness, and stiffness. Tactile sensing is especially crucial when areas are inaccessible for visual perception. With the rapid development in robotics, research in robotic control and force sensing has gained interest in the development of robots having surgeon-like tactile sensations. Besides, impedance sensing is another promising technology allowing the evaluation of the physiological state of the tissue, given that biological structures of the tissue cause impedance differences.

#### Clinical application

Bone drilling and milling are common surgical procedures in orthopedic surgeries. In manual bone-cutting procedures, surgeons use their tactile sensation to ensure a safe and accurate operation. Therefore, the majority of studies found in this category (36–43) proposed systems to compensate for the loss of the surgeon's tactile sensation in robotic surgery with force sensors and load cells (Table 5), whereas (44, 45) developed a custom-made tactile sensing probe using balloon expansion and impedance spectroscopy device allowing to differentiate between tissues in brain tumor resection surgery.

**Table 5:** General description of selected papers related to force, robotic, and impedance sensing.

Citation	Surgical Task	Sensor	Preprocessing	ML method	Material	Classes	Evaluation
Accini *et al.* 2016	bone drilling	custom-made mechatronic bone drilling tool	ramp-shaped position signal	N/A	bovine femoral shaft bone, chicken bone	breakthrough point	Acc. 100%
Al-Abdullah *et al.* 2019	robotic bone milling	6 DOF force sensor	feed rate/spindle speed measurement	Three-layer back propagation neural network	sawbones	cortical bone, 30pcf cancellous bone, 50pcf cancellous bone	N/A
Deng *et al.* 2013	robotic bone milling	force sensor	empirical mode decomposition, Hilbert transform, linear weighting combination, feature extraction (average amplitude/kurtosis, crest factor, average remaining)	SVM	pig scapula	outer cortical bone, cortical bone, cancellous bone, inner cortical bone	Rec. rate 86.7%–100%
Ho *et al.* 2018	robotic bone drilling	UR5 robot arm (feed rate, thrust force), current sensor	estimation of removal energy density		porcine bone	breakthrough point	N/A
Qu *et al.* 2021	robotic bone milling	UR5 robotic arm, six-axis force sensor, ultrasonic bone scalpel	wavelet transform denoising, data extracting, quick sorting, data removal, the mean of residual data estimation, normalization	BP NN	living pig spine	outer cortical bone layer, cancellous bone layer, inner cortical bone layer	Rec. rate 85%–100%
Tanaka *et al.* 2010	tumor resection	custom-made tactile sensing probe using balloon expansion	outer pressure of the balloon estimation	N/A	porcine brain	white matter, gray matter	N/A
Tian *et al.* 2014	robotic bone drilling	6-DOF force sensor	hybrid force feature extraction (the average value of force signal and force difference), recognition threshold as state recognition	N/A	sheep lumbar spine	initial state, outer cortical state, cancellous state, transitional state, inner cortical state	Acc. 100%
Torun *et al.* 2020	robotic bone drilling	load cell, DC drill motor, 6-DOF robot manipulator	motor current/control signal/instantaneous power of drill motor/ closed-loop speed error/ reference speed/ reference feed rate of robot/ thrust force measurement, low pass filter	KNN, Ensemble classifier	synthetic bone model, sheep femur	4-class, 9-class	Acc.98.2%–99.7%
Vadala *et al.* 2020	robotic bone drilling	load cell	the position-referenced average mechanica limpedance measurement from thrust force and feed rate	N/A	lumbar spine	pedicle cortical bone, pedicle cancellous bone, vertebral cancellous bone	N/A
Wang *et al.* 2015	robotic bone drilling	force sensor, optical tracking sensor	motor current, thrust force, deflection of a robot arm, rotate speed	SVM	pig scapula	the cortical, cortical-transit-cancellous, almost-break-cortical, cancellous states	Pre. 76.5%–96.3%, Re. 75.7%–96.4%
Wong *et al.* 2019	brain tumor resection	impedance spectroscopy device	resistance mapping reconstruction	N/A	rib-eye steak	fat, muscle fiber	Err. 2%

#### Technologies used

Robotic bone drilling and milling-related articles used force sensors (36, 37, 39, 40), load cells (41, 42), and electrical current sensors (38). Wang et al. (43) integrated an optical tracking system in the robotic setup to identify the milling states including cortical, cortical-transit-cancellous, almost-break-cortical, and cancellous states. Another method for active tactile sensing was proposed by Tanaka et al. (44), who used balloon expansion, which was brought in direct contact with the tissue and was expanded through the fluid, particularly biocompatible water. Based on pressure and volume changes, the stiffness and slimness of the underlying material in contact were derived. Another proof of concept study was performed by Wong et al. (45) using a bioimpedance spectroscopy device. They proposed that the resistance mapping reconstruction allowed the distinction between fat and muscle fibers.

#### Data processing

The approaches reported in Al-Abdullah et al. (36), Deng et al. (37), and Tian et al. (40) differentiated bone layers using force sensors based on feed rate and spindle speed measurements, Hilbert-Huang Transform based on empirical mode decomposition, and hybrid force feature extraction respectively. The work of Al-Abdullah et al. (36) applied an artificial neural network to synthetic bone boxes, whereas the hybrid force feature extraction method from Tian et al. (40) was based on thresholding. Data fusion opened the possibility to extract complementary multi-modal information in Al-Abdullah et al. (36), Ho et al. (38), Torun et al. (41), Vadala et al. (42), and Wang et al. (43). For example, Torun et al. (41) fed a KNN with motor current, control signal, instantaneous power of the drill motor, closed-loop speed error, reference speed, reference feed rate of the robot, and thrust force measurements to integrate an ensemble classifier.

#### Tissue classes and accuracy

The evaluation of the method in Torun et al. (41) classified 4 and 9 different states of robotic bone drilling and showed a 98.2% to 99.7% accuracy. The ground truth labels for drill bit states were obtained from video recordings and robot drill bit positions. Additionally, the data extracted from the sensors in Wang et al. (43) served as an input to SVM with 4 classes, namely cortical, cortical-transit-cancellous, almost-break-cortical, and cancellous states. The ground truth labels were acquired using an optical tracking system, which was used to track the relative position of the robotic arm with respect to anatomy. The assessment of the system on pig scapula demonstrated a 76.5%–96.3% precision and a 75.7%–96.4% recall. Based on the preliminary experiments in Tanaka et al. (44), the sensor was able to classify white matter from gray matter with the sensing time of 2s during ex-vivo porcine brain experiments.

#### Outlook

In addition to yet investigated fields of use like breach detection and tissue differentiation, force and impedance sensing could help spine surgeons to intraoperatively assess bone mineral density and quality of screw purchase or the need for cementation, respectively. This would help avoid revision surgery for mechanical failure at the implant-bone interface.

### Vibro-acoustic sensing

Auditory perception arises from sensing the vibrations in the physical world, whereas a vibration signal depicts information on the oscillations occurring during an equilibrium event. Therefore, vibration signals can characterize the differences between textures and detect the roughness of the surface.

#### Clinical application

In a surgical setup, information about the relative position between the instrument and the bone can be extracted from the cutting vibration. Specifically, the direct contact between the surgical tools as drilling and milling machines and bone induces vibration signals that differ with respect to the in-contact bone layer type. Vibration signals can be measured with accelerometers (47, 48) to safely navigate a bone milling procedure (Table 6). Surgeons also use their auditory perception to discern the distance between surgical tools and target anatomy. Inspired by this idea are microphone-based acoustic sensing approaches (49–56) used in bone drilling and milling.

**Table 6:** General description of selected paper related to vibro-acoustic sensing.

Citation	Surgical task	Sensor	Preprocessing	ML Method	Material	Classes	Evaluation
Dai *et al.* 2015	robotic bone milling	laser displacement sensor	median filter, WPT	Three-layer back propagation neural network	porcine spine	vertebrae, spinal cord, adjacent bony structure, muscle	Suc. rate 83%–100%
Dai *et al.* 2016	robotic bone milling	Single-axis accelerometer	median filter, WPT	SVM	porcine spine	vertebrae, spinal cord, adjacent bony structure, muscle	Suc. rate 95%–100%
Dai *et al.* 2017	robotic bone milling	free-field microphone, laser displacement sensor	bandpass filter based on WPT	self-organizing feature map	porcine spine	cortical bone, cancellous bone, annulus fibrosus, nothing	Suc. rate 85%–95%
Dai *et al.* 2018	robotic bone milling	free-field microphone, accelerometer	bandpass filter, correlation and covariance (sound pressure, acceleration signal), normalization	Three-layer back propagation neural network, PCA, LDA	porcine spine	cancellous, cortical, muscle, nothing	PPV 85–100%, NPV 95.2%–100%, Spec. 95.2%–100%, Sens. 84.2%–100%
Dai *et al.* 2021	robotic bone milling	Single-axis accelerometer	anti-aliasing filter, ADC converted, transformation to a square wave, serial to parallel converter	Hopfield Network	porcine spine	cortical bone, cancellous bone, mixture, nothing	PPV 83.6%–100%, NPV 96%–98.7%, Spec.94%–100%, Sens. 88%–98%
Feng *et al.* 2014	bone drilling	inertial measurement unit chip (gyroscope and 3-axial accelerometer)	variance, root mean square, mean absolute value, approximate entropy, continuous wavelet transforms + SVM	N/A	pig femur	cortical bone, cancellous bone	N/A
Ostler *et al.* 2020	robotic bone drilling	condenser microphone	log-melspectogram	CNN	porcine liver, muscle, fatty tissue	fascia, fat, idle, liver, muscle	Acc. 88.8%, Re. 89.10%, Pre. 89.04%, F1 89.07%
Seibold *et al.* 2021	bone drilling	self-made shielded piezo contact microphone	melspectrogram	ResNet18	hip sample	breakthrough, cortical bone	Sens. 84.38%–93.64%
Shevchik *et al.* 2021	bone cutting	non-contact acoustic microphone	WPT	Laplacian SVM, CNN, RDF, BNN	pork spare rib	skin, fat, muscle, bone	Acc. 89%–99%
Sun *et al.* 2014	robotic bone drilling	free-field microphone	FFT/ WPT, Exponential Mean Amplitude/ Hurst Exponent	N/A	porcine scapulae	outer cortical layer, inner cortical layer	Rec. rate 65.7%–88.6%
Torun *et al.* 2018	robotic bone drilling	sound recorder	PSD (Welsch method)	N/A	bone piecework	cancellous bone, cortical bone	Rec. rate 100%
Yu *et al.* 2015	bone milling	free-field microphone	WPT	N/A	porcine bone	cortical bone, cancellous bone	N/A

#### Technologies used

Acceleration sensing methods performed by Dai et al. (47, 48) tackled tissue classification during robotic bone drilling to make conclusions about material density. Several studies proposed to capture acoustic signals during surgical drilling and milling using free-field microphones (49, 53, 54), condenser microphones (50), contact microphones (51), and sound recorders (55). A more complex solution was proposed by Dai et al. (56), in which the authors combined a free-field microphone with an accelerometer.

#### Data processing

**Figure 3:**
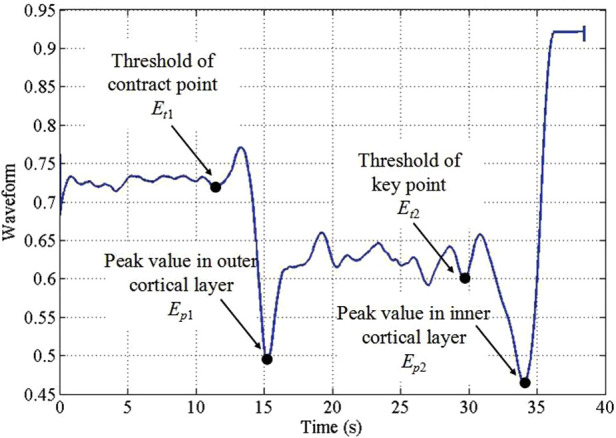
The exponential mean amplitude based state recognition from audio signal during robotic bone drilling (53).

Dai et al. (47) reported the use of wavelet packet transform feature extraction and SVM classification. In contrast, the more recent work reported in Dai et al. (48) implemented Hopfield networks after transforming the accelerometer signal to a square wave. Free-field microphone-based methods (49, 53, 54) extracted WPT features from porcine tissue samples. While Sun et al. (53) demonstrated that cortical and cancellous bone tissue can be classified by estimating the exponential mean amplitude (Figure 3), Dai et al. (49) introduced a self-organizing feature map method.

**Figure 4:**
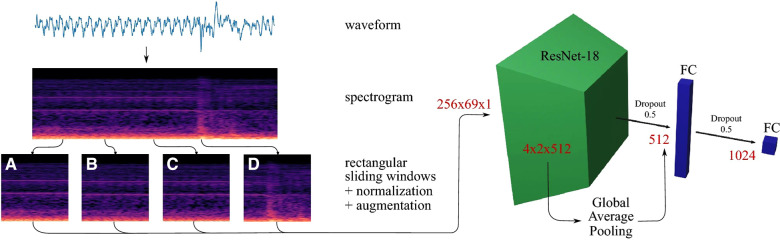
Data processing pipeline of a breakthrough detection method (51).

Methods found in Ostler et al. (50) and Seibold et al. (51) represented the state-of-the-art in machine learning, as they suggested classification by using deep learning architectures during bone drilling. Ostler et al. (50) extracted log-spectrograms acquired during tissue drilling. Seibold et al. (51) used mel-spectrograms of the audio signals with a ResNet18 network (Figure 4). In Dai et al. (56), after bandpass filtering of each signal source, correlation and covariance of sound pressure and acceleration signal were estimated. three-layer back propagation neural network, PCA, and LDA techniques were applied to the correlation and covariance measurements.

#### Tissue classes and accuracy

Dai et al. (47) classified vertebrae, spinal cord, adjacent bony structure, and muscle with a per class success rate of above 95%. Dai et al. (48) classified cortical bone, cancellous bone, and a mixture of cortical and cancellous bones with an 88%–98% sensitivity and a 94%–100% specificity. The ground truths were attained purely based on visual inspection of the samples. Dai et al. (49) classified cortical bone, cancellous bone, and annulus fibrosis with an 85%–95% success rate. In Ostler et al. (50), fascia, fat, porcine liver, and muscle were the output classes of the CNN and its accuracy, recall, precision, and F1-score were reported to be 88.8%, 89.10%, 89.04%, and 89.07% respectively. Their data were collected from experiments using 27 porcine specimens. Seibold et al. (51) reported that custom-made shielded piezo contact microphones were sufficiently sensitive to detect the cancellous and cortical bone drilling as well as the breakthrough events (transition from cortical to cancellous bone) from in-house cadaveric experiments. Their proposed deep-learning architecture showed a 93.64% breakthrough detection sensitivity.

#### Outlook

With its high specificity in classification of different bone tissue, vibro-acoustic sensing might be of particular promise in predicting pedicle breaches. Pedicle breaches can be predicted during the pedicle drilling step, when the drill or bone awl has close contact with cortical bone.

### Optical coherence tomography

Optical coherence tomography (OCT) is known as a non-invasive and radiation-free medical imaging technique capable of generating 2D or 3D images of biological tissues based on backscattered light measurement. It can be performed *in situ* and in real-time. Image resolution varies between 1 µm and 15 µm. However, a maximum imaging depth ranges between 2 mm and 3 mm (7).

#### Clinical application

All the extracted articles of this category (58–61) were related to tumor resection surgery and used similar OCT technology that reaches a maximum depth of 3 mm (Table 7).

**Table 7:** General description of selected papers related to optical coherence tomography.

Citation	Surgical Task	Sensor	Preprocessing	ML method	Material	Classes	Evaluation
Juarez-Chambi *et al.* 2019	brain tumor resection	OCT system	cropping, Canny edge detection, warping, peak detection, 2D entropy filtering	LR	brain tissue	non-cancerous tissue, glioma- infiltrated tissue	Sens. 90%, Spec. 82%
Möller *et al.* 2021	tumor resection	OCT system	local binary patterns/run-length analysis/Haralick's texture analysis/Laws texture energy measures estimation, median filtering, Canny edge detection, Otsu thresholding, cropping, normalization, PCA	SVM	lung, colon, breast tissues	vital tumor, healthy tissue, necrosis	Acc. 95.75%–99.10%
Lenz *et al.* 2018	brain tumor resection	OCT system	median filter, Canny edge detection, Otsu thresholding, cropping, normalization, PCA	SVM	brain tissue	healthy tissue, meningioma	Acc. 98%
Almog *et al.* 2020	stereotactic neurosurgery	Full-field swept-source OCT system	Maximum intensity projection, max pooling,Gray Level Co-Occurrence Matrix, Contrast/Correlation/Energy/homogeneity estimation	PCA	rat brain tissue	cortex, hippocampus, corpus callosum, striatum, thalamus	Acc. 75%

#### Technologies used

Möller et al. (59) used a commercially available spectral-domain OCT system (Thorlabs Ganymede, New Jersey, USA) with a 930 nm central wavelength, a 7 µm axial resolution, an 8 µm lateral resolution, a 1.4 refractive index, and the maximum penetration depth of 1.7 mm in air and 1.2 mm in brain tissue. Similarly, Lenz et al. (61) used a spectral-domain high-resolution OCT system (Thorlabs Ganydeme). It outstands the system in Möller et al. (59) owing to its 6 µm axial resolution, 1.36 refractive index, and maximal penetration depth of 2.7 mm in air and 2 mm in brain tissue. On the other hand, Almog et al. (60) introduced a full-field swept-source OCT system. Swept-source OCT is a type of OCT employing tunable laser sources to increase the sensitivity and speed of the system. Full-field acquisition capability of the OCT facilitates the 2D frame acquisition instead of point-by-point scanning. They developed a tailored endoscopic tip for the full-field swept-source OCT system to allow access to deep brain regions.

#### Data processing

The processing steps in Möller et al. (59) and Lenz et al. (61) involved median filtering, Canny edge detection, Otsu thresholding, normalization, and PCA. Möller et al. (59) and Lenz et al. (61) used SVM to identify if the tissue is healthy or tumorous. Patient C-scans in Almog et al. (60) were preprocessed to extract the texture features including the contrast, correlation, uniformity of energy, and homogeneity of an individual pixel with respect to neighboring pixels. Afterward, these estimates were fed to a PCA classification method.

#### Tissue classes and accuracy

Lenz et al. (61, 62) used brain tissue to discern meningioma from healthy tissue and showed a 98% classification accuracy. Möller et al. (60) implemented the classification algorithm to identify the vital tumor, healthy tissue, and necrosis in the lung, colon, and breast tissues. Their accuracy ranged between 95.75–99.10%. The method developed by Almog et al. (61) recognized different brain regions including cortex, hippocampus, corpus callosum, striatum, and thalamus. The classification accuracy reached 75%.

#### Outlook

As a non-ionizing high-resolution real-time medical imaging, optical coherence tomography could potentially aid with the classification of nervous tissue in the surgical treatment of intradural pathologies such as tumors or tethered cords.

### Endoscopic and microscopic imaging

Microscopic and endoscopic approaches are common MIS methods. Microscopic surgery is performed with high-powered microscopes that allow magnifying the field of view, whereas endoscopic surgery allows visualizing the hidden interior structures *via* an endoscopic tube with a small camera that is inserted into the incision area.

#### Clinical application

Studies using surgical endoscopes focused on spinal endoscopic surgeries (62) and percutaneous transforaminal endoscopic discectomies (63), where the identification of neural structures is critical. A surgical microscope was used in Haouchine et al. (64), Bai et al. (65), and Nercessian et al. (66) during craniotomy and microvascular decompression (Table 8).

**Table 8:** General description of selected paper related to microscopic and endoscopic imaging.

Citation	Surgical Task	Sensor	Preprocessing	ML Method	Material	Classes	Evaluation
Cui *et al.* 2019	spinal endoscopic surgery	surgical endoscope	N/A	YOLOv3	spine tissue	nerve, dura mater	Sens. 94.27%, Spec. 97.55%, Acc. 95.12%
Cui *et al.* 2021	percutaneous transforaminal endoscopic discectomy	surgical endoscope	N/A	YOLO v3	spine tissue	nerve, dura mater	Sens. 90.90%, Spec. 93.68%, Acc.92.29%, IoU 51.42%
Haouchine *et al.* 2015	craniotomy	surgical microscope	N/A	U-Net	brain tissue	vessel, parenchyma, background	IoU 0.647–0.744, Dice 0.786–0.852
Bai *et al.* 2021	microvascular decompression	surgical microscope	random horizontal flip, random scale cropping, random Gaussian blur, normalization	DeepLabv3+	brain tissue	trigeminal nerve, facial nerve, glossopharyngeal nerve, vagus nerve, anterior inferior cerebellar artery, posterior inferior cerebellar artery, petrosal vein	IoU 75.73%
Nercessian *et al.* 2021	craniotomy	surgical microscope	N/A	VGG-19 + U-Net	brain tissue	vessel, parenchyma, background	IoU 0.709, dice 0.822

#### Technologies used

A surgical endoscope was used in the studies by Cui et al. (62, 63), whereas Haouchine et al. (64), Bai et al. (65), and Nercessian et al. (66) relied on surgical microscopes. No detailed information about types and brands of the devices was given.

#### Data processing

Studies reported in Cui et al. (62, 63) used YOLO-v3 as the basic tissue classification algorithm, whereas Haouchine et al. (64) and Nercessian et al. (66) applied CNN architectures for semantic segmentation such as U-Net and VGG-19. A similar deep learning-based approach was used in Bai et al. (65), but the architecture was based on the semantic segmentation method known as DeepLabv3 + and was extended with the feature distillation block and the atrous spatial pyramid pooling modules.

#### Tissue classes and accuracy

Surgical endoscope-based studies (62, 63) identified nerve and dura, while surgical microscope-based studies (64, 66) classified vessel, parenchyma, and background to distinguish trigeminal nerve. Bai et al. (65) classified the facial nerve, glossopharyngeal nerve, vagus nerve, anterior inferior cerebellar artery, posterior inferior cerebellar artery, petrosal vein vessel, parenchyma, and background from microvascular decompression recordings. All the evaluation metrics reported in the endoscopic studies (62, 63) including sensitivity, accuracy, and specificity were reported to be on the order of 90%. The microscopic study by Bai et al. (65) reached a 75.73%.

#### Outlook

Spinal endoscopy and microsurgery already are indispensable pillars of spine surgery with continuous development. Currently, in addition to decompressions, lumbar fusion surgeries with interbody cages are also performed endoscopically. Here, a combination with other sensing technologies is of particular interest as haptic feedback is reduced.

### X-Ray imaging

X-ray imaging uses electromagnetic energy beams to produce images of various media based on the interaction of x-rays with matter. Two main phenomena happen during x-ray – matter interaction, namely absorption and scattering effects. Compton scatter, coherent scatter and photoelectric effects are the major effects that are considered in medical applications. The detection of x-rays can be accomplished either indirectly or directly. The (older) indirect x-ray detection method uses scintillators first to convert received radiation to light and then photodetectors to detect the generated light. Whereas the direct method is based on semiconductor detectors that convert x-ray photons directly into electrical signals. Although x-ray imaging is a widely used imaging technology in medicine, it is invasive due to the radiation exposure to the patient.

#### Clinical application

X-ray imaging is widely used in diagnosing tumors or bone fractures. Extracted papers (67, 68) are used in bone tumor diagnosis during tumor resection surgery (Table 9).

**Table 9:** General description of selected papers related to X-ray.

Citation	Surgical task	Sensor	Preprocessing	ML Method	Material	Classes	Evaluation
Furuo *et al.* 2021	bone tumor resection	X-ray	N/A	VGG16, ResNet152	knee bone	benign tumor, malignant tumor	Loss 1.07–1.31, Acc. 0.823–0.824, F1 0.784–0.790
Ho *et al.* 2019	bone tumor resection	X-ray	bone segmentation (bidirectional W-network)	VGG16, ResNet50, InceptionV3	knee bone	normal tissue, benign tumor, malignant tumor	Acc. 77.84% –86.93%,Pre. 77.15%–85.24%,Re. 77.94%–85.56%, F1 77.69%–85.29%

#### Technologies used

No technical details were provided.

#### Data processing

Furuo et al. (67) used deep learning architectures (VGG16 and Resnet152) directly on x-ray images. Ho et al. (46) proposed a more complex algorithm, which was an integration of a regenerative semi-supervised bidirectional W-network for segmenting femur, tibia, and fibula of knee region and classification step for a malignant tumor, benign tumor, and normal tissue with VGG16, ResNet50, and Inception-v3 networks.

#### Tissue classes and accuracy

The studies of Furuo et al. (68) and Ho et al. (46) acquired the x-ray images of the human knee region. Three classes were identified in Ho et al. (46) namely normal tissue, benign tumor, and malignant tumor. Furuo et al. (68) showed that VGG16 provided higher accuracy compared to Resnet 152 in terms of f1-score, specifically 0.790 vs. 0.784. Their accuracy was around 82%, while the method in Ho et al. (46) resulted in an accuracy range of 77%–87%.

#### Outlook

X-ray imaging probably has limited applicability for tissue classification in spinal surgery, as superimpositions particularly complicate the delineation of individual tissues in the spine. Research efforts are aimed more at 3-dimensional registration of the anatomy as a basis for low-radiation navigation.

## Discussion

### Clinical application

The clinical applications of reviewed methods can be categorized into two main groups. The first group concerns intraoperative tissue classification methods for brain tumor resection in neurosurgery. This group entails hyperspectral imaging (e.g., 9), spectroscopic sensing (e.g., 20), ultrasound imaging (e.g., 34), impedance sensing (e.g., 45), and OCT imaging (e.g., 61). Brain tumor resection is the most common type of treatment. It is critical to identify cancerous cells correctly, as resecting too much brain tissue can lead to neurological impairment of the patient. The current tumor assessment method in clinics involves pre-operative and post-operative imaging to identify the location of the tumor and evaluate the success of resection. However, pre-operative data can become outdated due to tissue manipulation or through dynamic changes in intracranial content which makes tissue detection essential.

The second group addresses intraoperative tissue classification in orthopedic surgery. Some of the surgical instruments used in orthopedic surgeries (e.g., scalpel, saw, burr, and drill) create forces, vibrations or even damage to the manipulated anatomical structure and surrounding tissues. Thus, real-time feedback to classify the tissue during orthopedic surgery is addressed using spectroscopic sensing (e.g., 31), force and robotic sensing (e.g., 41), vibro-acoustic sensing (e.g., 48), and microscopic and endoscopic imaging (e.g., 62). These technologies could be of particular value during orthopedic implant insertion as imminent fractures or bone breaches could be avoided, for instance when inserting a prosthetic stem in hip surgery or pre-drilling for pedicle screws in spine surgery. Especially for spine cases, such technical support is of notable value, where a reduced bone mineral density is regularly found and the transition between bone and surrounding soft tissues (e.g., spinal nerves) is critical.

### Technologies used

Among brain tumor detection-related studies, the technologies that were used to detect brain tumors (9) intraoperatively are mainly based on the optical properties of the tissue. These include technologies such as hyperspectral imaging (e.g., 9), Raman spectroscopy (e.g., 22), and optical coherence tomography [e.g., (64)]. Unlike other studies concerning the application of the VNIR hyperspectral push-broom camera (e.g., 9), Urbanos et al. (16) proposed the snapshot camera, which could be used in a surgical environment due to its small footprint. However, it should be noted that the major challenge till today in developing robust and real-time intraoperative tissue classification methods based on hyperspectral cameras is the large size of the hyperspectral images. This in turn leads to a longer data processing time and requires higher computational power. Additionally, ultrasound imaging was used to detect brain tumor (e.g., 34). It should be noted that the brain surface to be scanned with an ultrasound probe should be filled with a fluid – a saline solution. Besides, air should not be trapped in the resection cavity as it affects the visualization quality. Furthermore, surgical microscope-based tissue classification during craniotomy was proposed in Haouchine et al. (64) and Nercessian et al. (66). They particularly targeted the segmentation of cortical vessels located at the brain surface by claiming that it can serve as an additional information resource to resolve the issue of brain shift caused by craniotomy.

The majority of the work related to orthopedic surgery based their tissue classification methods on the biomechanical properties of the bone during bone cutting, milling, and drilling processes. Such work formed on the accelerometer (e.g., 47), microphone (e.g., 53), force sensor (e.g., 37), load cell (e.g., 42) and laser technologies (e.g., 10). Experiments were mainly done with robotic arms (e.g., 38, 39) since the major area of application is the tissue classification during robotic drilling or milling to compensate for the tactile or auditory perception of the human. In addition, ultrasound was added to the orthopedic sensing category, as it can be used to differentiate bone from soft tissues.

Overall, based on current analysis, integration of sensors [e.g., a fiber laser and non-contact microphones (49), a free-field microphone and an accelerometer (56), VNIR and NIR cameras (14), etc.] can be noted as the recent trend in intraoperative tissue classification. Potentially, the investigated imaging modalities like hyperspectral imaging or optical coherence tomography could be of clinical use due to their real-time performance and ease of integration into existing visual systems such as surgical microscopes.

### Data processing

Recent work on tissue classification using hyperspectral imaging used CNNs after image calibration, noise filtering, band reduction, and normalization steps (e.g., 17, 18). Similarly, data processing before applying a CNN (e.g., U-Net) from Raman spectrometer involved noise filtering, band reduction, and normalization steps (e.g., 32). On the other hand, within the force, tactile, and robotic control-based sensing group, the majority of algorithms were developed with the aid of classical machine learning approaches such as SVM (e.g., 37) and KNN (e.g., 41). Similarly, the majority of studies concerning vibro-acoustic sensing classified the tissue type using a three-layer back propagation neural network (e.g., 10) and SVM (e.g., 47). Except for the microphone sensing methods that applied additionally CNNs to the spectrogram (e.g., 50). Imaging information-based studies including x-ray and surgical endoscopes used similarly computer vision algorithms such as YOLO v3 (e.g., 63) and VGG (e.g., 68).

Overall, the application of various deep learning approaches facilitates the possibility of interpreting the data in a more robust and precise fashion compared with conventional signal processing methods. However, the development of a deep-learning approach requires access to large amounts of annotated data. The reviewed methods providing tissue classification algorithms based on imaging methods (microscope e.g., 65, x-ray e.g., 68, ultrasound e.g., 12) created the required training datasets by manually labeling done by medical specialists. This manual labeling requires meticulous attention and can result in human error. Whereas, Raman spectroscopy-related studies (e.g., 24) were labeled with histopathological evaluation, which is generally considered to be more accurate. The tactile sensing methods (43) in bone tissue type identification were also accompanied by computer navigation systems, enabling labelling of the signals based on the relative position between milling tool and anatomy. Since optical tracking systems are known as highly accurate in spatial localization, they enable accurate labeling of the bone tissue type. On the other hand, hyperspectral imaging data of the reviewed methods was labeled with semi-automatic software (e.g., 9). In addition to accurate labeling, the size and heterogeneity of the dataset have to be sufficiently large to achieve a high performance in the classification.

### Tissue classes and accuracy

Hyperspectral imaging-based methods classify healthy tissue, tumor, blood vessels, and dura mater in brain tumor resection surgery (e.g., 9). They showed accuracy in the order of 84%–85% with a VNIR hyperspectral push-broom camera (17). Although the deep learning integration with the snapshot camera reached an accuracy of only 49%–60% in Urbanos et al. (16), it is still a promising technology for intraoperative tissue classification in MIS owing to its small size. On the other hand, ultrasound and optical coherence tomography-based tissue classification methods in brain resection surgery targeted the identification of tumors (e.g., glioblastoma, solitary brain metastases) and healthy tissue. Their performance in binary classification tasks was found to be promising. For example, the OCT-based classifier achieved 98% accuracy in Lenz et al. (61) and the ultrasound system-based approach reached 90% accuracy in Ritschel et al. (35). Both methods were integrated with traditional machine learning methods such as SVM among extracted papers. Their performance may be further improved by more complex networks in the future such as deep learning networks and perhaps increase the number of classes.

The majority of the work related to orthopedic surgery is aimed at the classification of different bone tissues. They mainly focused on discriminating cortical bone, cancellous bone and the transition state from cortical to cancellous. However, the data fusion approach presented in Torun et al. (41) identified more states (4 and 9) with 98.2%–99.7% accuracy. This provides support and evidence for the potential of data fusion in tissue classification, which was also verified in the vibro-acoustic sensing group, where the integration of accelerometer with free-field microphone achieved 84.2%–100% accuracy

## Conclusion

Both orthopedic and neurological studies in this current review paper showed that a combination of sensors from different modalities (e.g., vibro-acoustic and force sensing) can provide much-needed redundancy and complement the overall information on tissue types, which in turn can help in making more comprehensive decisions. Thus, a sensor fusion deep-learning enabled approach is one of the most promising research directions that could substantially enhance existing technologies for intraoperative tissue classification.

## Data Availability

The original contributions presented in the study are included in the article/Supplementary Material, further inquiries can be directed to the corresponding author/s.

## References

[1] Kalfas IH. Machine vision navigation in spine surgery. Front Surg. (2021) 8:1–7. 10.3389/fsurg.2021.640554 33738298 PMC7960759

[2] Camacho JE Usmani MF Strickland AR Banagan KE Ludwig SC. The use of minimally invasive surgery in spine trauma: a review of concepts. J Spine Surg. (2019) 5(S1):S91–100. 10.21037/jss.2019.04.13 31380497 PMC6626750

[3] Gelalis ID Paschos NK Pakos EE Politis AN Arnaoutoglou CM Karageorgos AC Accuracy of pedicle screw placement: a systematic review of prospective in vivo studies comparing free hand,fluoroscopy guidance and navigation techniques. Eur Spine J. (2012) 21(2):247–55. 10.1007/s00586-011-2011-3 21901328 PMC3265579

[4] Farshad M Aichmair A Gerber C Bauer DE. Classification of perioperative complications in spine surgery. Spine J. (2020) 20(5):730–6. 10.1016/j.spinee.2019.12.013 31877388

[5] Overley SC Cho SK Mehta AI Arnold PM. Navigation and robotics in spinal surgery: where are we now? Clin Neurosurg. (2017) 80(3):S86–99. 10.1093/neuros/nyw077 28350944

[6] Joskowicz L Hazan EJ. Computer aided orthopaedic surgery: incremental shift or paradigm change? Med Image Anal. (2016) 33:84–90. 10.1016/j.media.2016.06.036 27407004

[7] Nevzati E Marbacher S Soleman J Perrig WN Diepers M Khamis A Accuracy of pedicle screw placement in the thoracic and lumbosacral spine using a conventional intraoperative fluoroscopy-guided technique: A national neurosurgical education and training center analysis of 1236 consecutive screws. World Neurosurgery. (2014) 82:866–871.e2. 10.1016/j.wneu.2014.06.023 24954252

[8] Sipos E Tebo S Zinreich S Long D Brem H. In vivo accuracy testing and clinical experience with the ISG Viewing Wand. Neurosurgery. (1996) 39(1):194–202. 10.1097/00006123-199607000-00048 8805161

[9] Fabelo H Halicek M Ortega S Sarmiento R Szolna A Morera J Surgical aid visualization system for glioblastoma tumor identification based on deep learning and in-vivo hyperspectral images of human patients. SPIE-Intl Soc Optical Eng. (2019) 10951:35. 10.1117/12.2512569 PMC6708415 31447494

[10] Dai Y Xue Y Zhang J. Tissue discrimination based on vibratory sense in robot-assisted spine surgery. Proceedings - IEEE International Conference on Robotics and Automation. Institute of Electrical and Electronics Engineers Inc. (2015). p. 4717–22.

[11] Chen H Xu W Broderick N. Raman spectroscopy for minimally invasive spinal nerve detection. M2VIP 2016 - Proceedings of 23rd International Conference on Mechatronics and Machine Vision in Practice. (2017).

[12] Carson T Ghoshal G Cornwall GB Tobias R Schwartz DG Foley KT. Artificial intelligence-enabled, real-time intraoperative ultrasound imaging of neural structures within the psoas. Spine. (2021) 46(3):E146–52. 10.1097/BRS.0000000000003704 33399436 PMC7787186

[13] Page MJ McKenzie JE Bossuyt PM Boutron I Hoffmann TC Mulrow CD The PRISMA 2020 statement: an updated guideline for reporting systematic reviews. BMJ. (2021) 372:1–9. 10.1136/bmj.n71 PMC8005924 33782057

[14] Leon R Fabelo H Ortega S Piñeiro JF Szolna A Hernandez M VNIR–NIR hyperspectral imaging fusion targeting intraoperative brain cancer detection. Sci Rep. (2021) 11(1):1–12. 10.1038/s41598-021-99220-0 PMC8490425 34608237

[15] Fabelo H Ortega S Ravi D Kiran BR Sosa C Bulters D Spatio-spectral classification of hyperspectral images for brain cancer detection during surgical operations. PLoS One. (2018) 13(3):1–27. 10.1371/journal.pone.0193721 29554126 PMC5858847

[16] Urbanos G Martín A Vázquez G Villanueva M Villa M Jimenez-Roldan L Supervised machine learning methods and hyperspectral imaging techniques jointly applied for brain cancer classification. Sensors. (2021) 21(11):1–29. 10.3390/s21113827 34073145 PMC8199064

[17] Fabelo H Halicek M Ortega S Shahedi M Szolna A Piñeiro JF Deep learning-based framework for In Vivo identification of glioblastoma tumor using hyperspectral images of human brain. Sensors. (2019) 19(4):1–25. 10.3390/s19040920 PMC6412736 30813245

[18] Manni F van der Sommen F Fabelo H Zinger S Shan C Edström E Hyperspectral imaging for glioblastoma surgery: improving tumor identification using a deep spectral-spatial approach. Sensors. (2020) 20(23):1–20. 10.3390/s20236955 PMC7730670 33291409

[19] Andrews R Mah R Jeffrey S Aghevli A Freitas K Guerrero M Multimodality tissue identification for neurosurgery: the NASA smart probe project. Proceedings - Applied Imagery Pattern Recognition Workshop; 2000 Jan; (2000). p. 153–7. 10.1109/AIPRW.2000.953618

[20] Broadbent B Brusatori MA Koya K Scarpace LM Kalkanis SN Auner GW. Fresh brain tissue diagnostics using Raman spectroscopy in humans. IEEE International Conference on Electro Information Technology; 2018 May. (2018). 394–8.

[21] Cakmakci D Karakaslar EO Ruhland E Chenard MP Proust F Piotto M Machine learning assisted intraoperative assessment of brain tumor margins using HRMAS NMR spectroscopy. PLoS Comput Biol. (2020) 16(11):1–14. 10.1371/journal.pcbi.1008184 33175838 PMC7682900

[22] Hollon TC Lewis S Pandian B Niknafs YS Garrard MR Garton H Rapid intraoperative diagnosis of pediatric brain tumors using stimulated Raman histology. Cancer Res. (2018) 78(1):278–89. 10.1158/0008-5472.CAN-17-1974 29093006 PMC5844703

[23] Hollon TC Pandian B Adapa AR Urias E Save A Khalsa SSS Near real-time intraoperative brain tumor diagnosis using stimulated Raman histology and deep neural networks. Nat. Med. (2020) 26:52–8. 10.1038/s41591-019-0715-9 31907460 PMC6960329

[24] Livermore LJ Isabelle M Bell IM Edgar O Voets NL Stacey R Raman Spectroscopy to differentiate between fresh tissue samples of glioma and normal brain: a comparison with 5-ALA-induced fluorescence-guided surgery. J Neurosurg. (2021) 135(2):469–79. https://thejns.org/doi/abs/10.3171/2020.5.JNS20376 10.3171/2020.5.JNS20376 33007757

[25] Riva M Sciortino T Secoli R D’amico E Moccia S Fernandes B Glioma biopsies classification using Raman spectroscopy and machine learning models on fresh tissue samples. Cancers. (2021) 13(5):1–14. 10.3390/cancers13051073 PMC7959285 33802369

[26] Uckermann O Galli R Tamosaityte S Leipnitz E Geiger KD Schackert G Label-free delineation of brain tumors by coherent anti-stokes Raman scattering microscopy in an orthotopic mouse model and human glioblastoma. PLoS One. (2014) 9(9):1–9. 10.1371/journal.pone.0107115 PMC4159970 25198698

[27] Zhou Y Liu CH Wu B Yu X Cheng G Zhu K Optical biopsy identification and grading of gliomas using label-free visible resonance Raman spectroscopy. J Biomed Opt. (2019) 24(09):1. 10.1117/1.JBO.24.9.095001 PMC6997631 31512439

[28] Kamen A Sun S Wan S Kluckner S Chen T Gigler AM Automatic tissue differentiation based on confocal endomicroscopic images for intraoperative guidance in neurosurgery. BioMed Res Int. (2016) 2016:1–8. 10.1155/2016/6183218 27127791 PMC4835625

[29] Kenhagho H Canbaz F Gomez Alvarez-Arenas TE Guzman R Cattin P Zam A. Machine learning-based optoacoustic tissue classification method for Laser osteotomes using an air-coupled transducer. Lasers Surg Med. (2021) 53(3):377–89. 10.1002/lsm.23290 32614077

[30] Gunaratne R Monteath I Goncalves J Sheh R Ironside CN Kapfer M Machine learning classification of human joint tissue from diffuse reflectance spectroscopy data. Biomed Opt Express. (2019) 10(8):3889. 10.1364/BOE.10.003889 31452982 PMC6701523

[31] Laws SG Souipas S Davies BL Baena FRY. Toward automated tissue classification for markerless orthopaedic robotic assistance. IEEE Trans Med Robot Bionics. (2020) 2(4):537–40. 10.1109/TMRB.2020.3031716

[32] Puustinen S Alaoui S Bartczak P Bednarik R Koivisto T Dietz A Spectrally tunable neural network-assisted segmentation of microneurosurgical anatomy. Front Neurosci. (2020) 14:1–9. 10.3389/fnins.2020.00640 32694976 PMC7339939

[33] Shen B Zhang Z Shi X Cao C Zhang Z Hu Z Real-time intraoperative glioma diagnosis using fluorescence imaging and deep convolutional neural networks. Eur J Nucl Med Mol Imaging. (2021) 48:3482–92. 10.1007/s00259-021-05326-y 33904984 PMC8440289

[34] Cepeda S García-García S Arrese I Fernández-Pérez G Velasco-Casares M Fajardo-Puentes M Comparison of intraoperative ultrasound B-mode and strain elastography for the differentiation of glioblastomas from solitary brain metastases. An automated deep learning approach for image analysis. Front Oncol. (2021) 10(February):1–11. 10.3389/fonc.2020.590756 PMC7884775 33604286

[35] Ritschel K Pechlivanis I Winter S. Brain tumor classification on intraoperative contrast-enhanced ultrasound. Int J Comput Assist Radiol Surg. (2015) 10(5):531–40. 10.1007/s11548-014-1089-6 24956998

[36] Al-Abdullah KI Lim CP Najdovski Z Yassin W. A model-based bone milling state identification method via force sensing for a robotic surgical system. Int J Med Robot Comp Assiste Surg. (2019) 15(3):1–16. 10.1002/rcs.1989 30721570

[37] Deng Z Zhang H Guo B Jin H Zhang P Hu Y Hilbert-Huang transform based state recognition of bone milling with force sensing. 2013 IEEE International Conference on Information and Automation, ICIA; 2013 Aug. (2013). p. 937–42.

[38] Ho D Li T Meng QH. Bone drilling breakthrough detection via energy-based signal. Proceedings of the Annual International Conference of the IEEE Engineering in Medicine and Biology Society, EMBS; 2018 Jul. (2018). p. 1809–12. 10.1109/EMBC.2018.8512621 30440746

[39] Qu H Geng B Chen B Zhang J Yang Y Hu L Force perception and bone recognition of vertebral lamina milling by robot-assisted ultrasonic bone scalpel based on backpropagation neural network. IEEE Access. (2021) 9:52101–12. 10.1109/ACCESS.2021.3069549

[40] Tian W Han X Liu B Liu Y Hu Y Han X A robot-assisted surgical system using a force-image control method for pedicle screw insertion. PLoS One. (2014) 9(1):1–9. 10.1371/journal.pone.0086346 PMC3899254 24466043

[41] Torun Y Öztürk A. A new breakthrough detection method for bone drilling in robotic orthopedic surgery with closed-loop control approach. Ann Biomed Eng. (2020) 48(4):1218–29. 10.1007/s10439-019-02444-5 31897891

[42] Vadalà G Ambrosio L Portaccio I Accoto D Russo F De Salvatore S Validation of a novel smart drilling system to monitor bone impedance during transpedicular screw placement: a pilot study. In: Castoldi F Peretti GM Marmotti A Mangiavini L, editors. Congress of the Italian orthopaedic research society 2019 (2020) Torino, Italy: Journal of Biological Regulators & Homeostatic Agents 34(4 Supplement 3). p. 251. 33261286

[43] Wang Y Deng Z Sun Y Yu B Zhang P Hu Y State detection of bone milling with multi-sensor information fusion. 2015 IEEE International Conference on Robotics and Biomimetics, IEEE-ROBIO; 2015 (2015). p. 1643–8.

[44] Tanaka Y Yu Q Doumoto K Sano A Hayashi Y Fujii M Development of a real-time tactile sensing system for brain tumor diagnosis. Int J Comput Assist Radiol Surg. (2010) 5(4):359–67. 10.1007/s11548-010-0426-7 20414734

[45] Wong SS Ekanayake J Liu Y Constandinou TG. An impedance probing system for real-Time intraoperative brain tumour tissue discrimination. BioCAS 2019 - Biomedical Circuits and Systems Conference, Proceedings; 2019 (2019–22).

[46] Accini F Díaz I Gil JJ. Using an admittance algorithm for bone drilling procedures. Comput Methods Programs Biomed. (2016) 123:150–8. 10.1016/j.cmpb.2015.10.003 26516110

[47] Dai Y Xue Y Zhang J. Milling state identification based on vibration sense of a robotic surgical system. IEEE Trans Ind Electron. (2016) 63(10):6184–93. 10.1109/TIE.2016.2574981

[48] Dai Y Xue Y Zhang J. Human-inspired haptic perception and control in robot-assisted milling surgery. IEEE Trans Haptics. (2021) 14(2):359–70. 10.1109/TOH.2020.3029043 33044941

[49] Dai Y Xue Y Zhang J Li J. Biologically-inspired auditory perception during robotic bone milling. Proceedings - IEEE International Conference on Robotics and Automation. (2017). p. 1112–6.

[50] Ostler D Seibold M Fuchtmann J Samm N Feussner H Wilhelm D Acoustic signal analysis of instrument–tissue interaction for minimally invasive interventions. Int J Comput Assist Radiol Surg. (2020) 15(5):771–9. 10.1007/s11548-020-02146-7 32323212 PMC7261275

[51] Seibold M Maurer S Hoch A Zingg P Farshad M. Real-time acoustic sensing and artificial intelligence for error prevention in orthopedic surgery. Sci Rep. (2021) 11:1–11. 10.1038/s41598-021-83506-4. 33597615 PMC7889943

[52] Shevchik S Nguendon Kenhagho H Le-Quang T Faivre N Meylan B Guzman R Machine learning monitoring for laser osteotomy. J Biophotonics. (2021) 14(4):1–11. 10.1002/jbio.202000352 33369169

[53] Sun Y Jin H Hu Y Zhang P Zhang J. State recognition of bone drilling with audio signal in robotic orthopedics surgery system. Proceedings - IEEE International Conference on Robotics and Automation (Iros). (2014). p. 3538–43.

[54] Yu D Yuan X Jianxun Z. State identification based on sound analysis during surgical milling process. 2015 IEEE International Conference on Robotics and Biomimetics, IEEE-ROBIO 2015;0172. (2015): p. 1666–9.

[55] Torun Y Ozturk A Hatipoglu N Oztemur Z. Breakthrough detection for orthopedic bone drilling via power spectral density estimation of acoustic emission. 2018 Electric Electronics, Computer Science, Biomedical Engineerings’ Meeting, EBBT (2018). p. 1–5.

[56] Dai Y Xue Y Zhang J. Bioinspired integration of auditory and haptic perception in bone milling surgery. IEEE/ASME Trans Mechatronics. (2018) 23(2):614–23. 10.1109/TMECH.2018.2804950

[57] Feng C Wong K Meng MQH Ren H. Drilling pattern analysis of femur bone based on inertial measurement unit signal. 2014 IEEE International Conference on Information and Automation, ICIA 2014. (2014): 841–5.

[58] Juarez-Chambi RM Kut C Rico-Jimenez JJ Chaichana KL Xi J Campos-Delgado DU AI-assisted in situ detection of human glioma infiltration using a novel computational method for optical coherence tomography. Clin Cancer Res. (2019) 25(21):6329–38. 10.1158/1078-0432.CCR-19-0854 31315883 PMC6825537

[59] Möller J Bartsch A Lenz M Tischoff I Krug R Welp H Applying machine learning to optical coherence tomography images for automated tissue classification in brain metastases. Int J Comput Assist Radiol Surg. (2021) 16(9):1517–26. 10.1007/s11548-021-02412-2 34053010 PMC8354973

[60] Almog IF der CF Senova S Fomenko A Gondard E Sacher WD Full-field swept-source optical coherence tomography and neural tissue classification for deep brain imaging. J Biophotonics. (2020) 13(2):1–11. 10.1002/jbio.201960083 31710771 PMC7065632

[61] Lenz M Krug R Dillmann C Stroop R Gerhardt NC. Automated differentiation between meningioma and healthy brain tissue based on optical coherence tomography ex vivo images using texture features. J Biomed Opt. (2018) 23(07):1. 10.1117/1.JBO.23.7.071205 29484876

[62] Cui P Guo Z Xu J Li T Shi Y Chen W Tissue recognition in spinal endoscopic surgery using deep learning. 2019 IEEE 10th International Conference on Awareness Science and Technology, iCAST 2019 - Proceedings. (2019).

[63] Cui P Shu T Lei J Chen W. Nerve recognition in percutaneous transforaminal endoscopic discectomy using convolutional neural network. Med Phys. (2021) 48(5):2279–88. 10.1002/mp.14822 33683736

[64] Haouchine N Nercessian M Juvekar P Golby A Frisken S. Cortical vessel segmentation for neuronavigation using vesselness-enforced deep neural networks. IEEE Trans Med Robot Bionics. (2021) 14(8):1–5. 10.1109/TMRB.2021.3122337

[65] Bai R Jiang S Sun H Yang Y Li G. Deep neural network-based semantic segmentation of microvascular decompression images. Sensors. (2021) 21(4):1–16. 10.3390/s21041167 PMC7915571 33562275

[66] Nercessian M Haouchine N Juvekar P Frisken S Golby A. Deep cortical vessel segmentation driven by data augmentation with neural image analogy. Proceedings - International Symposium on Biomedical Imaging. (2021-April). p. 721–4.

[67] Furuo K Morita K Hagi T Nakamura T Wakabayashi T. Automatic benign and malignant estimation of bone tumors using deep learning. 2021 5th IEEE International Conference on Cybernetics, CYBCONF 2021; (2021). p. 30–3.

[68] Ho NH Yang HJ Kim SH Jung ST Joo SD. Regenerative semi-supervised bidirectional w-network-based knee bone tumor classification on radiographs guided by three-region bone segmentation. IEEE Access. (2019) 7:154277–89. 10.1109/ACCESS.2019.2949125

[69] Shapey J Xie Y Nabavi E Bradford R Saeed SR Ourselin S Intraoperative multispectral and hyperspectral label-free imaging: a systematic review of in vivo clinical studies. J Biophotonics. (2019) 12:1–13. 10.1002/jbio.201800455 30859757 PMC6736677

